# Resilience in the Face of Trauma: A Pediatric Case of Traumatic Pneumatocele

**DOI:** 10.7759/cureus.63450

**Published:** 2024-06-29

**Authors:** Rafla Al Kayyali, Majid Alteneiji

**Affiliations:** 1 General Pediatrics, Tawam Hospital, Al Ain, ARE; 2 Pulmonology, Tawam Hospital, Al Ain, ARE

**Keywords:** pulmonary, cyst, pulmonary cavitations, traumatic pulmonary pseudocyst, traumatic pneumatocele

## Abstract

Traumatic pneumatocele (TP) is a rare complication that can develop in the lungs following a traumatic event. These lesions are sometimes mistaken for congenital airway malformations. Multiple theories exist to explain the pathophysiology of this condition. This case study presents the clinical and radiological findings of a seven-year-old patient diagnosed with pneumatocele on thoracic imaging after a motor vehicle accident. A detailed evaluation of the patient's medical history and imaging led to the diagnosis of traumatic pneumatocele. This uncommon presentation, if not well understood, may lead to unnecessary interventions and significant anxiety for patients and their families. Given its rarity, awareness and a high index of suspicion are essential for accurate diagnosis and appropriate management.

## Introduction

Traumatic pneumatoceles (TP), though rare, have been described in the literature. These cavitary lesions, filled with air and situated within the pulmonary parenchyma without an epithelial lining, commonly follow blunt chest trauma [1]. Other names have been used to describe them, such as traumatic lung cysts, pulmonary cavitation, cavitating hematoma, and traumatic pulmonary pseudocysts [1,2]. Pneumatoceles are often asymptomatic but can be present with symptoms such as cough, dyspnea, chest pain, hemoptysis, and respiratory distress; however, these symptoms are nonspecific and may frequently occur in the context of blunt thoracic trauma [1,2]. Different mechanisms of injury are involved, most commonly motor vehicle accidents, followed by falls and sports [1,2]. Only a few cases described may lead to life-threatening hemoptysis [3]. The appearance of TP on radiographic images, showing single or multiple cavities within the lung filled with air, blood, or fluid, suggests a more serious injury than a simple contusion [4]. Associated complications may include pneumothorax and pneumomediastinum. Accurate diagnosis is crucial to avoid unnecessary procedures, as TP typically resolves without surgical intervention.

## Case presentation

A six-year-old male with a prior diagnosis of epilepsy presented to the hospital following a rear-impact motor vehicle accident. The patient was seated in the back seat without restraints and was not ejected during the car accident. Upon arrival at the hospital, he was anxious and complained of generalized body pain. On physical examination, he was found to be tachypneic, otherwise hemodynamically stable. A primary survey indicated decreased air entry and crackles on the right side of the chest, with a Glasgow Coma Scale score of 15/15. A complete secondary survey showed that he had lower limb abrasions. An Extended Focused Assessment with Sonography in Trauma (eFAST) indicated no abnormalities.

**Figure 1:**
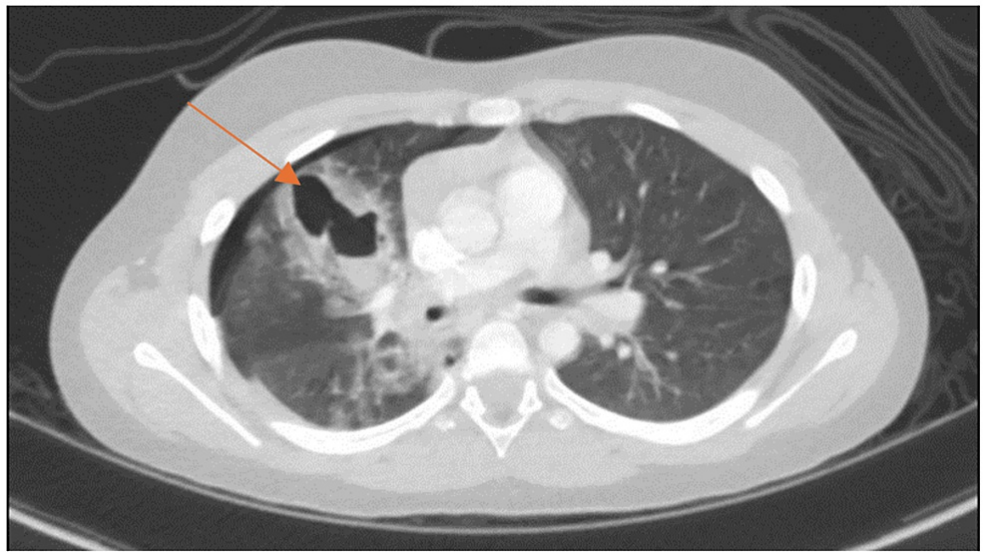
Transverse section CT of the thorax. Right-sided pneumothorax and patchy inhomogeneous airspace opacification in the right lung and multiple cavities with air-fluid levels. The arrow pointing to the cavity with the air-fluid level.

**Figure 2:**
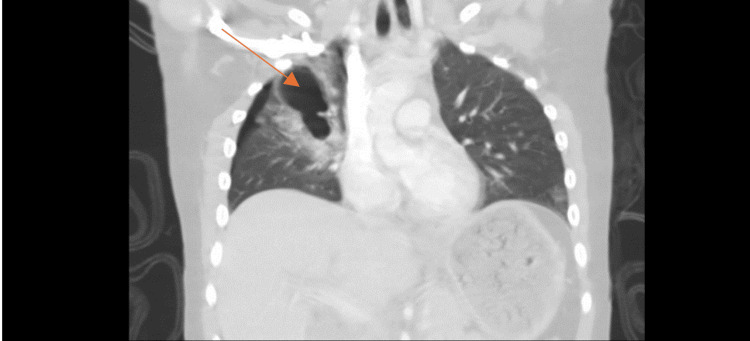
Coronal section CT of the thorax. Right-sided pneumothorax and patchy inhomogeneous airspace opacification in the right lung and multiple cavities with the air-fluid level. The arrow pointing to the cavity and opacification.

A CT scan of the chest demonstrated a patchy inhomogeneous airspace opacification in the right lung with multiple cavities with the air-fluid level, suggestive of TP, alongside a grade 2 liver injury (Figure 1 and Figure 2). Blood tests were within normal ranges, with a mild elevation in liver enzymes correlating with the CT findings.

**Figure 3:**
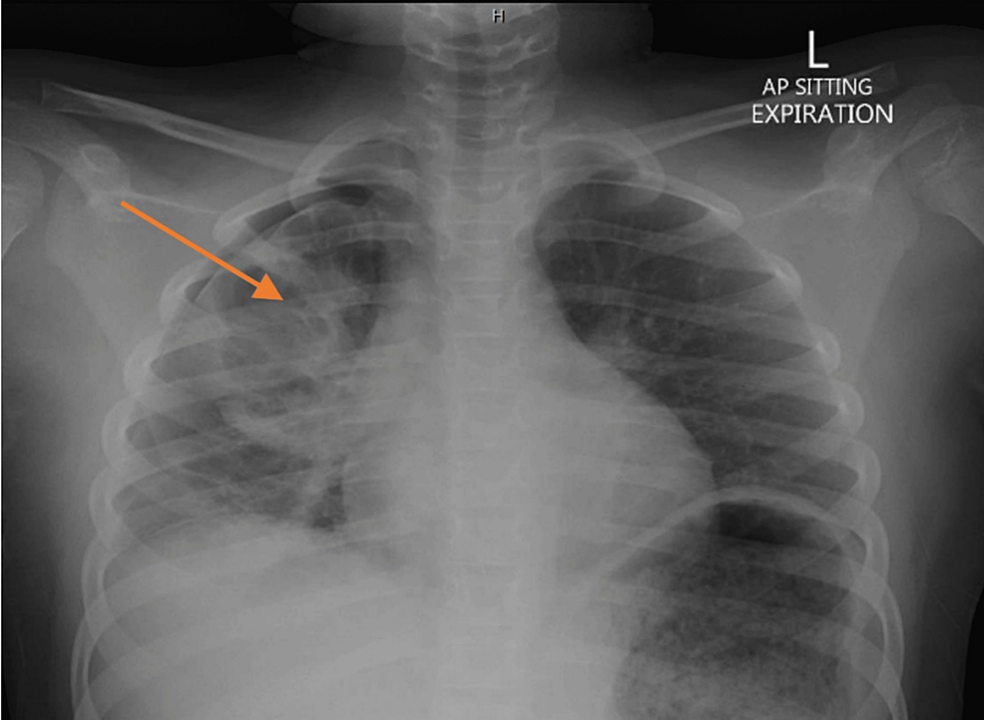
Chest X-ray. Small right-sided pneumothorax and right upper zone opacity. The arrow pointing to the right upper zone opacity.

**Figure 4:**
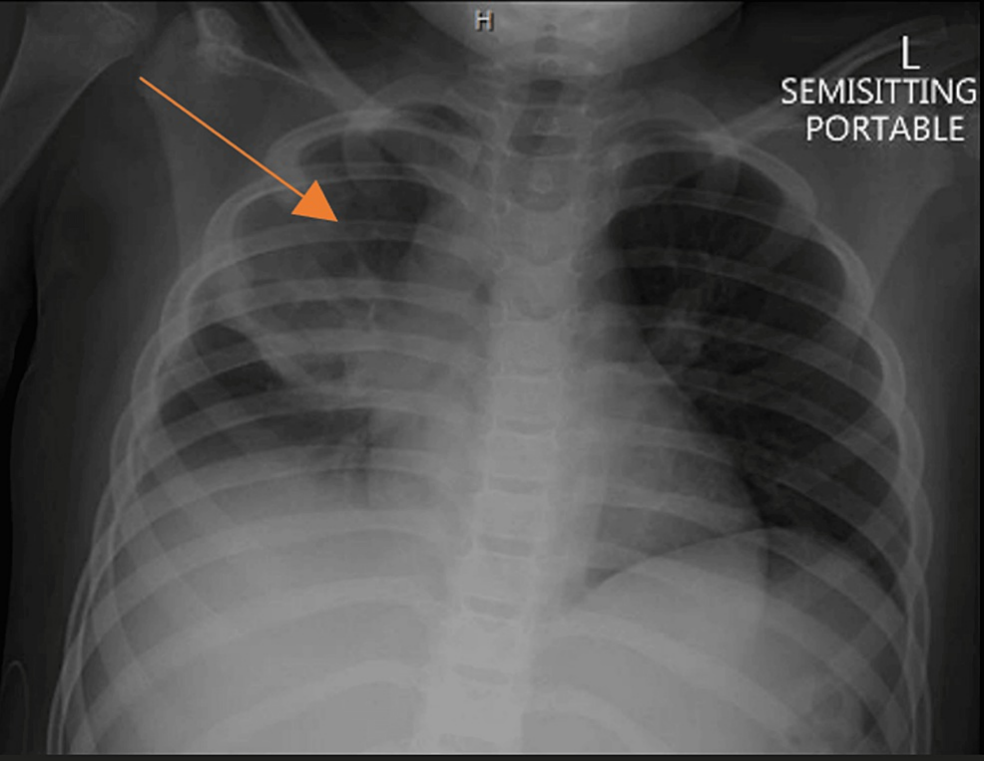
Chest X-ray. The improvement of the right pneumothorax with a stable upper zone opacity. The arrow pointing to the right upper zone opacity.

**Figure 5:**
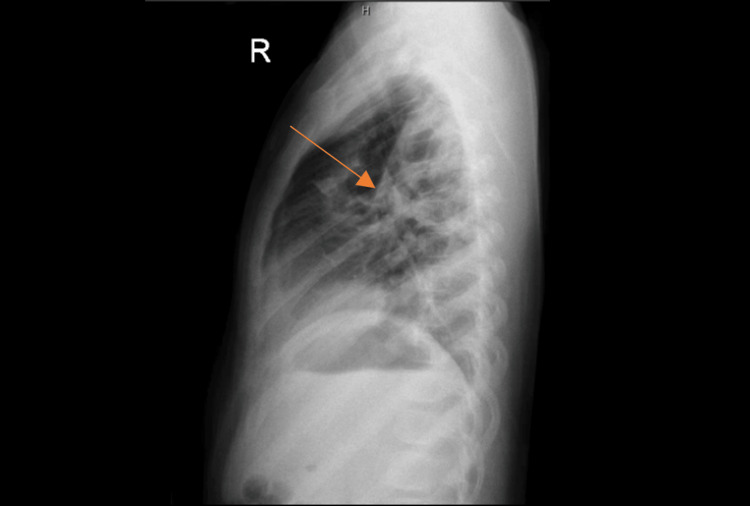
Chest X-ray. Cavitary lesions with air-fluid levels in the upper lung. The arrow pointing to the air-fluid level.

The patient was admitted to the pediatric intensive care unit (PICU) for observation; he required supplemental oxygen and remained stable. Subsequent chest X-rays identified a small right-sided pneumothorax and an upper zone opacity on the right side (Figure 3), which improved on subsequent imaging nine hours later (Figure 4). However, the right upper lobe opacity persisted with a demonstration of the air-fluid level (Figure 5).

During his hospital stay, the patient was closely monitored in the PICU. His clinical condition improved, and oxygen supplementation was gradually reduced until he was able to maintain oxygen saturation on room air. The patient was discharged on the sixth day following admission.

**Figure 6:**
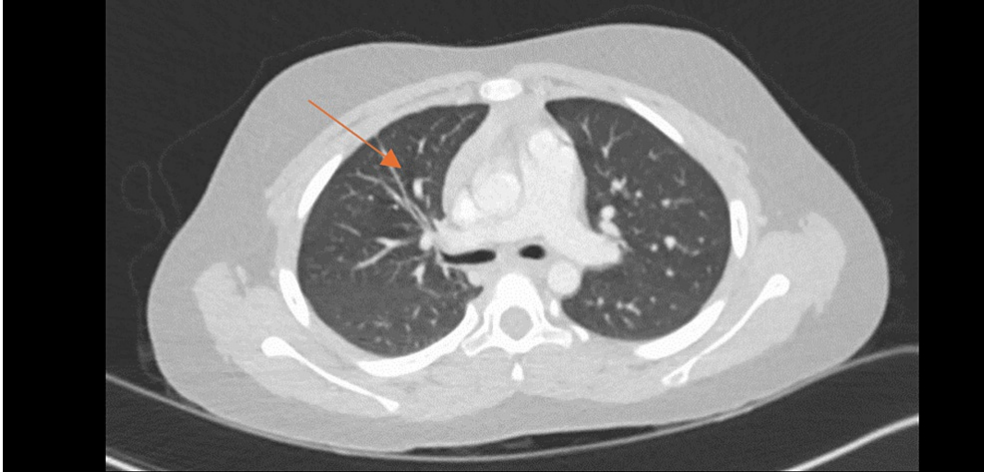
Repeat CT scan seven months later showing the resolution of the previously seen inhomogeneous opacification/cysts (transverse view). The arrow pointing to the resolution of the previously seen inhomogeneous opacification/cavity.

**Figure 7:**
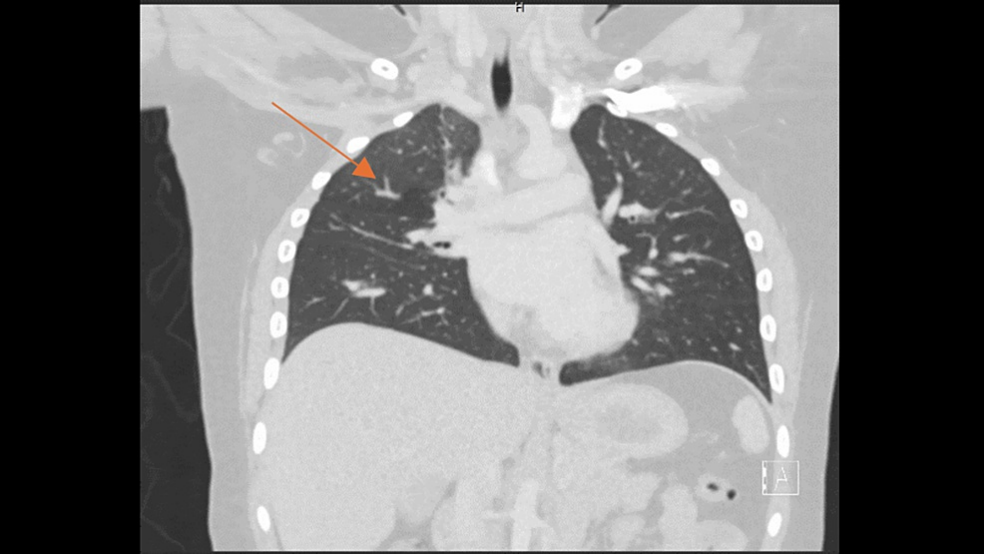
Repeat CT scan seven months later showing the resolution of the previously seen inhomogeneous opacification/cysts (frontal view). The arrow pointing to the resolution of the previously seen inhomogeneous opacification/cavity.

At follow-up, the patient continued to improve and did not experience any respiratory symptoms. A follow-up CT of the thorax seven months later demonstrated an improvement in the previously mentioned right-sided upper lung zone opacity and the resolution of the lung cavities (Figure 6 and Figure 7), confirming the diagnosis of a traumatic pneumatocele.

## Discussion

Traumatic pneumatocele (TP) manifests in less than 4% of pediatric cases, and its clinical relevance remains to be fully elucidated [5]. Predominantly affecting children and adolescents, TP incidence is higher in males, potentially due to a greater likelihood of involvement in motor vehicle accidents [2,6].

The susceptibility of the pediatric population to TP might be attributed to two factors: 1) more flexible ribs and 2) increased fragility of the lung parenchyma. The pathogenesis of traumatic pneumatoceles (TP) is hypothesized to occur in a two-step process. Initially, blunt chest trauma results in increased intrabronchial pressures (barotrauma), resulting in a ruptured parenchyma. Subsequently, due to the elastic recoil of the thoracic cage in a pediatric patient, a negative pressure differential occurs, resulting in cavities that are filled with air, fluid, or blood [6,7]. Following the blunt trauma from the accident, our patient was lodged between the front and rear seats following the accident, which may have accentuated air leaks to the cavities due to airway obstruction.

The presentation of TP depends on the severity of the blunt chest trauma. In mild cases, the patient might be asymptomatic. In more severe cases, it can present with cough, dyspnea, chest pain, hemoptysis, fever, and leukocytosis. Fortunately, our patient had mild symptoms with minimal oxygen need. In addition, TP might appear with other associated injuries, termed "complicated TP." Such injuries include diaphragmatic rupture, esophageal injuries, or, like in our case, a pneumothorax. It has been reported that TP greater than 4 cm in diameter is associated with complications such as infections, hematoceles, and failure to respond to conservative management [7,8].

The diagnosis of TP is primarily radiological. CT scans offer a superior sensitivity of 96% over chest X-rays at 24% sensitivity [1,3]. The images may show single or multiple lung cysts, which may be located anywhere in the lungs. However, the lung apices are usually spared [5]. It is crucial to differentiate the radiological findings of TP from other causes, for example, congenital lung malformations (i.e., congenital pulmonary adenomatoid malformation {CPAM}), recurrent pneumonia, tuberculosis, and pneumatoceles associated with pneumonia. In our case, a ruptured congenital lung malformation was initially considered. Luckily, a previous chest X-ray performed prior to the injury showed no abnormalities, which was further confirmed by a repeat CT scan of the chest seven months later. There was no prior history of chronic cough, fever, hemoptysis, weight loss, or recurrent chest infections, ruling out an infectious etiology.

The management of TP depends on the severity of associated injuries. In the absence of other complications, TP resolves on its own. The time it requires for full resolution varies between individuals, ranging from weeks to months [4]. Generally, it is usually a benign condition with no reported long-term complications [9]. In this case, a repeat CT scan showed the resolution of the TP. Complicated cases, however, usually require surgical intervention and take longer to resolve fully. One study described the time required for the resolution of an uncomplicated TP as around three months, whereas complicated TP usually takes longer, with a mean time of 145 days, depending on the associated injuries [10].

## Conclusions

Traumatic pneumatoceles represent a rare but significant condition within the pediatric population, necessitating accurate early recognition and management to avoid unnecessary interventions. This case highlights the effectiveness of careful observation and follow-up in managing TP, showcasing the condition's potential for complete resolution without invasive procedures.
